# Comparison of five tumor regression grading systems for gastric adenocarcinoma after neoadjuvant chemotherapy: a retrospective study of 192 cases from National Cancer Center in China

**DOI:** 10.1186/s12876-017-0598-5

**Published:** 2017-03-14

**Authors:** Yuelu Zhu, Yongkun Sun, Shangying Hu, Yong Jiang, Jiangying Yue, Xuemin Xue, Lin Yang, Liyan Xue

**Affiliations:** 1 Department of Pathology and Resident Training Base, National Cancer Center/Cancer Hospital, Chinese Academy of Medical Sciences and Peking Union Medical College, Beijing, China; 2 Department of Medical Oncology, National Cancer Center/Cancer Hospital, Chinese Academy of Medical Sciences and Peking Union Medical College, Beijing, China; 3 Department of Cancer Epidemiology, National Cancer Center/Cancer Hospital, Chinese Academy of Medical Sciences and Peking Union Medical College, Beijing, China; 4 Department of Pathology, Esophageal Carcinoma Hospital of Linzhou, Linzhou, Henan China

**Keywords:** Gastric cancer, Neoadjuvant chemotherapy, Tumor regression grading

## Abstract

**Background:**

Neoadjuvant chemotherapy has been increasingly practiced on gastric cancer (GC), and histological evaluation to predict outcome is urgent in clinical practice. There are five classic tumor regression grading (TRG) systems, including Mandard-TRG system, the Japanese Gastric Cancer Association (JGCA)-TRG system, College of American Pathologists (CAP)-TRG system, China-TRG system and Becker-TRG system.

**Methods:**

Totally, 192 patients of gastric adenocarcinoma (including adenocarcinoma of the esophagogastric junction) treated by neoadjuvant chemotherapy and surgery were evaluated using the above five TRG systems. The clinicopathological characteristics were also assessed. The correlation among TRG systems, clinicopathological characteristics and prognosis were analyzed.

**Results:**

All the five TRG systems were significantly correlated with differentiation, postsurgical T category, postsurgical N category, American Joint Committee on Cancer (AJCC) stage, lymph-vascular invasion, perineural invasion, as well as tumor size. All the five TRG systems were statistically significant in univariate Cox survival analysis. However, only postsurgical T category, postsurgical N category and R0 resection were independent in multivariate Cox survival analysis. The tight correlation between the TRG systems and other characteristics such as postsurgical stage might affect the independent prognostic role of the TRG systems. As compared with other TRG systems, the hazard ratio of no/slightly response in both Mandard TRG system and JGCA TRG system revealed higher hazard of death and disease progression than that of severe response when using univariate Cox survival analysis. The median survival time of complete response and nearly complete response were much longer than that of partial response, all classified by Mandard-TRG system. This could help clinicians predict prognosis more reasonably than JGCA-TRG which does not have the category of nearly complete response.

**Conclusion:**

We recommend Mandard-TRG system for GC after neoadjuvant chemotherapy due to its better prediction of prognosis.

## Background

In 2012, 951,000 new diagnoses of gastric cancer (GC) and 723,000 GC deaths were calculated worldwide, accounting for 6.8% of all cancer incidence and 8.8% of all cancer mortality, respectively [1]. At present, GC is the second common cancer in China, while approximately 679,100 new cancer cases and 498,000 related deaths were estimated in China in 2015 [2]. The most promising curative treatment for GC is surgical resection. However, this treatment alone is not enough for curing advanced GC because of poor long-term outcome, thus a multimodality therapy is required. Nowadays, neoadjuvant chemotherapy has been increasingly used to prolong patient survival worldwide [3], therefore an effective histopathological evaluation method predicting patient prognosis is urgently needed in clinical practice. So far, four tumor regression grading (TRG) systems were presented by several studies. Mandard et al. proposed a five-tiered TRG system in esophageal carcinoma which has been used widely in digestive malignancy [4]. The Japanese Gastric Cancer Association (JGCA) suggested a different five-tiered grading system specifically for GC [5]. College of American Pathologists (CAP) recommended a simplified four-tiered grading system based on Mandard-TRG system [6]. In China, a three-tiered grading system has been used for solid malignancies to evaluate the extent of therapy-related tumor regression [7]. In recent years, Becker et al. recommended a four-tiered grading system for GC based on large number of patients and long-term follow-up [8, 9]. For the purpose of further evaluation of the relationship between TRG and prognosis, we retrospectively collected 192 patients in our hospital. We used the above five TRG systems to assess the pathological response respectively, trying to select a better histopathological evaluation system.

## Methods

### Patients

Between January 2007 and August 2013, 192 patients with locally advanced gastric adenocarcinoma (including adenocarcinoma of the esophagogastric junction) underwent gastrectomy in National Cancer Center/Cancer Hospital, Chinese Academy of Medical Sciences and Peking Union Medical College. All the patients received neoadjuvant chemotherapy previously. The treatment strategies were not uniform, and the drugs most used included oxaliplatin, cisplatin, docetaxel, 5-fluorouracil and Tegafur Gimeracil Oteracil Potassium Capsule. There were 133 patients who received additional adjuvant chemotherapy after surgery. All cases had only one primary gastric tumor, except one patient, who had two primary lesions located at the fundus and antrum, separately. The tumor located at the fundus was evaluated in our study for its relatively higher T category. There were 139 male and 53 female, and the age ranged 31–77 years old (median age, 55 years old).

### Follow-up

The mortality data was mainly gathered from clinical archives, or via telephone and mail. After treatment, patients were evaluated every 3 months for the first 2 years, and subsequently every 6 months for the following 3 years, and then annually according to institutional policy. Information of recurrence was updated every time the patients came for follow-up visits. The time of overall-survival (OS) was calculated from the first day of the neoadjuvant chemotherapy to the day when death occurred or to the last follow-up (September 2015). The time of progression-free-survival (PFS) was calculated from the first day of the neoadjuvant chemotherapy to the day when progression happened, death occurred or to the last follow-up (September 2015). Eleven patients were lost to follow-up. The median follow-up time was 31 months (4.1–95.3 months). Three patients, who died as the result of surgery complications, and 8 patients, whose follow-up time after surgery were less than 3 months, were excluded from the survival analysis.

### Assessment of the specimens

If obvious residual carcinoma was identified macroscopically, the tumor specimen was sampled for at least 1 block per centimeter. If the maximum of residue was less than 3 cm or only areas of scar existed, the whole suspected lesion was submitted completely for histological examination. Four-micrometer sections were stained with hematoxylin and eosin. The average of slides per residual tumor was 9.7 (3–77), while the median number of the examined lymph nodes per case was 24 (3–58).

### Clinicopathological characteristics

The clinicopathological features include tumor location, tumor size, histological differentiation, Laurén classification, lymph-vascular invasion (LVI) and perineural invasion (PNI). The tumor staging was based on the American Joint Committee on Cancer (AJCC) cancer staging 7th edition [10]. All the slides were reviewed by three experienced pathologists (YZ, JY and LX). In case of a disagreement about diagnosis, three pathologists reviewed the slides on a multi-headed microscope and reached a consensus diagnosis.

### Tumor regression grading systems

The criteria of the five TRG systems are shown in Table 1.

**Table 1:** Criteria of five tumor regression grading systems

TRG system	Grade	Description
Mandard-TRG	1	No residual cancer
	2	Rare residual cancer cells
	3	Fibrosis outgrowing residual cancer
	4	Residual cancer outgrowing fibrosis
	5	Absence of regressive changes
JGCA-TRG	0	No evidence of effect
	1a	Viable tumor cells occupy more than 2/3 of the tumorous area
	1b	Viable tumor cells remain in more than 1/3 but less than 2/3 of the tumorous area
	2	Viable tumor cells remain in less than 1/3 of the tumorous area
	3	No viable tumor cells remain
CAP-TRG	0	No viable cancer cells (complete response)
	1	Single cells or small groups of cancer cells (moderate response)
	2	Residual cancer outgrown by fibrosis (minimal response)
	3	Minimal or no tumor killed or extensive residual cancer (poor response)
China-TRG	Severe response	Tumor cells completely disappear or very few highly regressive residue exist with obvious scarring and varying inflammation
	Moderate response	Most tumor cells degenerate and necrosis with obvious stroma fibrosis and inflammation
	Mild response	Absence of or slight necrosis and degeneration of tumor cells accompanied by mild stroma fibrosis and inflammation
Becker-TRG	1a	No residual tumor/tumor bed
	1b	<10% residual tumor/tumor bed
	2	10–50% residual tumor/tumor bed
	3	>50% residual tumor/tumor bed

### Statistical analysis

Correlations between tumor regression evaluation systems and clinicopathological characteristics were performed by χ2 tests. And 2-sided *p*-values < 0.05 were considered statistically significant. The survival curve and median survival time were calculated using Kaplan-Meier method and log rank test. The univariate analysis of survival was evaluated by univariate Cox regression analysis. The statistic significant factors in univariate analysis were assessed by backward stepwise multivariate Cox regression analysis. Variables with a *p*-value of <0.05 were retained, and variables with a p-value of >0.10 were removed. All statistics were performed by SPSS 16.0 software (SPSS Inc, Chicago, IL).

## Results

### Clinicopathological characteristics

All the 192 patients received radical surgery. Forty-nine patients underwent proximal subtotal gastrectomy. Eighty-eight patients underwent distal subtotal gastrectomy. Fifty-three patients underwent total gastrectomy. Two patients underwent residual gastrectomy, both of which underwent distal gastrectomy previously due to severe ulcer. Sixty-three patients underwent D1 lymphadenectomy and 129 underwent D2 lymphadenectomy. The examined numbers of the removed lymph nodes per case ranged from 3 to 58. One-hundred and fifty-nine patients got R0 resection (82.8%). Twenty-one patients had local unresectable residues detected macroscopically by the surgeons while 12 had microscopically positive margins. Forty-four patients had adenocarcinoma of the esophagogastric junction (22.9%), 71 had tumors located at proximal stomach (37.0%), while 77 had tumors located at distal stomach (40.1%). We used maximal diameter of residual tumor/tumor bed to describe the size of tumor, which was divided into three groups [8]: <4.5 cm (*n* = 108, 56.3%), 4.5–8 cm (*n* = 64, 33.3%) and >8 cm (*n* = 20, 10.4%). The clinicopathological characteristics were tabulated in Tables 2, 3, 4, 5 and 6.

**Table 2:** Relationships between clinicopathological characteristics and pathological response evaluated by Mandard-TRG system

Characteristics	Total cases (*n* = 192), no. (%)	Mandard-TRG					*P* value
		1 (*n* = 11), no. (%)	2 (*n* = 23), no. (%)	3 (*n* = 40), no. (%)	4 (*n* = 78), no. (%)	5 (*n* = 40), no. (%)	
Gender							0.039
Male	139 (72.4)	10 (7.2)	20 (14.4)	29 (20.9)	48 (34.5)	32 (23)	
Female	53 (27.6)	1 (1.9)	3 (5.7)	11 (20.8)	30 (56.6)	8 (15.1)	
Age							0.867
< 55y	89 (46.4)	6 (6.7)	10 (11.2)	17 (19.1)	39 (43.8)	17 (19.1)	
≥ 55y	103 (53.6)	5 (4.9)	13 (12.6)	23 (22.3)	39 (37.9)	23 (22.3)	
Location							0.351
Esophagogastric junction	44 (22.9)	1 (2.3)	4 (9.1)	8 (18.2)	20 (45.5)	11 (25)	
Proximal gastric	71 (37)	7 (9.9)	8 (11.3)	15 (21.1)	23 (32.4)	18 (25.4)	
Distal gastric	77 (40.1)	3 (3.9)	11 (14.3)	17 (22.1)	35 (45.5)	11 (14.3)	
Maximal diameter of tumor bed							0.004
< 4.5 cm	108 (56.2)	9 (8.3)	20 (18.5)	20 (18.5)	44 (40.7)	15 (13.9)	
4.5–8 cm	64 (33.3)	1 (1.6)	3 (4.7)	17 (26.6)	27 (42.2)	16 (25)	
> 8 cm	20 (10.4)	1 (5)	0 (0)	3 (15)	7 (35)	9 (45)	
Histological differentiation							0.003
Well-moderate	45 (23.4)	4 (8.9)	12 (26.7)	9 (20)	16 (35.6)	4 (8.9)	
Poor	147 (76.6)	7 (4.8)	11 (7.5)	31 (21.1)	62 (42.2)	36 (24.5)	
Laurén classification							0.240
Intestinal	77 (40.1)	4 (5.2)	14 (18.2)	14 (19.2)	28 (36.4)	15 (19.5)	
Diffuse	73 (38)	7 (9.6)	5 (6.8)	10 (23.8)	33 (45.2)	14 (19.2)	
Mixed	42 (21.9)	0 (0)	4 (9.5)	15 (35.7)	17 (40.5)	11 (26.2)	
LVI							<0.001
Negative	94 (49)	11 (11.7)	19 (20.2)	28 (29.8)	26 (27.7)	10 (10.6)	
Positive	98 (51)	0 (0)	4 (4.1)	12 (12.2)	52 (53.1)	30 (30.6)	
PNI							<0.001
Negative	76 (39.6)	11 (14.5)	22 (28.9)	13 (17.1)	23 (30.3)	7 (9.2)	
Positive	116 (60.4)	0 (0)	1 (0.9)	27 (23.3)	55 (47.4)	33 (28.4)	
AJCC ypT category							<0.001
0	11 (5.7)	11 (100)	0 (0)	0 (0)	0 (0)	0 (0)	
1	20 (10.4)	0 (0)	12 (60)	5 (25)	3 (15)	0 (0)	
2	23 (12)	0 (0)	5 (21.7)	6 (26.1)	12 (52.2)	0 (0)	
3	57 (29.7)	0 (0)	5 (8.8)	18 (31.6)	25 (43.9)	9 (15.8)	
4	81 (42.2)	0 (0)	1 (1.2)	11 (13.6)	38 (46.9)	31 (38.3)	
AJCC ypN category							<0.001
0	55 (28.6)	9 (16.4)	13 (23.6)	17 (30.9)	10 (18.2)	6 (10.9)	
1	36 (18.8)	0 (0)	6 (16.7)	7 (19.4)	16 (44.4)	7 (19.4)	
2	50 (26)	0 (0)	3 (6)	10 (20)	32 (64)	5 (10)	
3	51 (26.6)	2 (3.9)	1 (2)	6 (11.8)	20 (39.2)	22 (43.1)	
AJCC stage							<0.001
0	9 (4.7)	9 (100)	0 (0)	0 (0)	0 (0)	0 (0)	
1	24 (12.5)	0 (0)	14 (58.3)	7 (29.2)	2 (8.3)	1 (4.2)	
2	53 (27.6)	2 (3.8)	6 (11.3)	14 (26.4)	21 (39.6)	10 (18.9)	
3	106 (55.2)	0 (0)	3 (2.8)	19 (17.9)	55 (51.9)	29 (27.4)	
R0 resection							0.185
Yes	159 (82.8)	11 (6.9)	20 (12.6)	35 (22)	64 (40.3)	29 (18.2)	
No	33 (17.2)	0 (0)	3 (9.1)	5 (15.2)	14 (42.4)	11 (33.3)	
Adjuvant chemotherapy							0.766
Not received	59 (30.7)	3 (5.1)	6 (10.2)	10 (16.9)	25 (42.4)	15 (25.4)	
Received	133 (69.3)	8 (6)	17 (12.8)	30 (22.6)	53 (39.8)	25 (18.8)

**Table 3:** Relationships between clinicopathological characteristics and pathological response evaluated by JGCA-TRG system

Characteristics	Total cases (*n* = 192), no. (%)	JGCA -TRG					*P* value
		3 (*n* = 11), no. (%)	2 (*n* = 63), no. (%)	1b (*n* = 45), no. (%)	1a (*n* = 33), no. (%)	0 (*n* = 40), no. (%)	
Gender							0.097
Male	139 (72.4)	10 (7.2)	48 (34.5)	30 (21.6)	19 (13.7)	32 (23)	
Female	53 (27.6)	1 (1.9)	15 (28.3)	15 (28.3)	14 (26.4)	8 (15.1)	
Age							0.711
< 55y	89 (46.4)	6 (6.7)	26 (29.2)	24 (27)	16 (18)	17 (19.1)	
≥ 55y	103 (53.6)	5 (4.9)	37 (35.9)	21 (20.4)	17 (16.5)	23 (22.3)	
Location							0.287
Esophagogastric junction	44 (22.9)	1 (2.3)	12 (27.3)	10 (22.7)	10 (22.7)	11 (25)	
Proximal gastric	71 (37)	7 (9.9)	23 (32.4)	15 (21.1)	8 (11.3)	18 (25.4)	
Distal gastric	77 (40.1)	3 (3.9)	28 (36.4)	20 (26)	15 (19.5)	11 (14.3)	
Maximal diameter of tumor bed							<0.001
< 4.5 cm	108 (56.2)	9 (8.3)	40 (37)	27 (25)	17 (15.7)	15 (13.9)	
4.5–8 cm	64 (33.3)	1 (1.6)	21 (32.8)	14 (21.9)	12 (18.8)	16 (25)	
> 8 cm	20 (10.4)	1 (5)	2 (10)	4 (20)	4 (20)	9 (45)	
Histological differentiation							0.060
Well-moderate	45 (23.4)	4 (8.9)	21 (46.7)	10 (22.2)	6 (13.3)	4 (8.9)	
Poor	147 (76.6)	7 (4.8)	42 (28.6)	35 (23.8)	27 (18.4)	36 (24.5)	
Laurén classification							
Intestinal	77 (40.1)	4 (5.2)	29 (37.7)	18 (23.4)	11 (14.3)	15 (19.5)	
Diffuse	73 (38)	7 (9.6)	20 (27.4)	16 (21.9)	16 (21.9)	14 (19.2)	
Mixed	42 (21.9)	0 (0)	14 (33.3)	11 (26.2)	6 (14.3)	11 (26.2)	
LVI							<0.001
Negative	94 (49)	11 (11.7)	46 (48.9)	19 (20.2)	8 (8.5)	10 (10.6)	
Positive	98 (51)	0 (0)	17 (17.3)	26 (26.5)	25 (25.5)	30 (30.6)	
PNI							<0.001
Negative	76 (39.6)	11 (14.5)	35 (46.1)	14 (18.4)	9 (11.8)	7 (9.2)	
Positive	116 (60.4)	0 (0)	28 (24.1)	31 (26.7)	24 (20.7)	33 (28.4)	
AJCC ypT category							<0.001
0	11 (5.7)	11 (100)	0 (0)	0 (0)	0 (0)	0 (0)	
1	20 (10.4)	0 (0)	17 (85)	1 (5)	2 (10)	0 (0)	
2	23 (12)	0 (0)	11 (47.8)	6 (26.1)	6 (26.1)	0 (0)	
3	57 (29.7)	0 (0)	24 (42.1)	16 (28.1)	8 (14)	9 (15.8)	
4	81 (42.2)	0 (0)	11 (13.6)	22 (27.2)	17 (21)	31 (38.3)	
AJCC ypN category							<0.001
0	55 (28.6)	9 (16.4)	30 (54.5)	5 (9.1)	5 (9.1)	6 (10.9)	
1	36 (18.8)	0 (0)	13 (26)	11 (30.6)	5 (13.9)	7 (19.4)	
2	50 (26)	0 (0)	13 (26)	21 (42)	11 (22)	5 (10)	
3	51 (26.6)	2 (3.9)	7 (13.7)	8 (15.7)	12 (23.5)	22 (43.1)	
AJCC stage							<0.001
0	9 (4.7)	9 (100)	0 (0)	0 (0)	0 (0)	0 (0)	
1	24 (12.5)	0 (0)	21 (87.5)	1 (4.2)	1 (4.2)	1 (4.2)	
2	53 (27.6)	2 (3.8)	20 (37.7)	12 (22.6)	9 (17)	10 (18.9)	
3	106 (55.2)	0 (0)	22 (20.8)	32 (30.2)	23 (21.7)	29 (27.4)	
R0 resection							0.046
Yes	159 (82.8)	11 (6.9)	55 (34.6)	40 (25.2)	24 (15.1)	29 (18.2)	
No	33 (17.2)	0 (0)	8 (24.2)	5 (15.2)	9 (27.3)	11 (33.3)	
Adjuvant chemotherapy							0.676
Not received	59 (30.7)	3 (5.1)	16 (27.1)	13 (22)	12 (20.3)	15 (25.4)	
Received	133 (69.3)	8 (6)	47 (35.3)	32 (24.1)	21 (15.8)	25 (18.8)

**Table 4:** Relationships between clinicopathological characteristics and pathological response evaluated by CAP-TRG system

Characteristics	Total cases (*n* = 192), no. (%)	CAP-TRG				*P* value
		0 (*n* = 11), no. (%)	1 (*n* = 23), no. (%)	2 (*n* = 40), no. (%)	3 (*n* = 118), no. (%)	
Gender						0.134
Male	139 (72.4)	10 (7.2)	20 (14.4)	29 (20.9)	80 (57.6)	
Female	53 (27.6)	1 (1.9)	3 (5.7)	11 (20.8)	38 (71.7)	
Age						0.880
< 55y	89 (46.4)	6 (6.7)	10 (11.2)	17 (19.1)	56 (62.9)	
≥ 55y	103 (53.6)	5 (4.9)	13 (12.6)	23 (22.3)	62 (60.2)	
Location						0.525
Esophagogastric junction	44 (22.9)	1 (2.3)	4 (9.1)	8 (18.2)	31 (70.5)	
Proximal gastric	71 (37)	7 (9.9)	8 (11.3)	15 (21.1)	41 (57.7)	
Distal gastric	77 (40.1)	3 (3.9)	11 (14.3)	17 (22.1)	46 (59.7)	
Maximal diameter of tumor bed						0.013
< 4.5 cm	108 (56.2)	9 (8.3)	20 (18.5)	20 (18.5)	59 (54.6)	
4.5–8 cm	64 (33.3)	1 (1.6)	3 (4.7)	17 (26.6)	43 (67.2)	
> 8 cm	20 (10.4)	1 (5)	0 (0)	3 (15)	16 (80)	
Histological differentiation						0.002
Well-moderate	45 (23.4)	4 (8.9)	12 (26.7)	9 (20)	20 (44.4)	
Poor	147 (76.6)	7 (4.8)	11 (7.5)	31 (21.1)	98 (66.7)	
Laurén classification						0.143
Intestinal	77 (40.1)	4 (5.2)	14 (18.2)	14 (19.2)	47 (64.4)	
Diffuse	73 (38)	7 (9.6)	5 (6.8)	10 (23.8)	28 (66.7)	
Mixed	42 (21.9)	0 (0)	4 (9.5)	15 (35.7)	23 (54.8)	
LVI						<0.001
Negative	94 (49)	11 (11.7)	19 (20.2)	28 (29.8)	36 (38.3)	
Positive	98 (51)	0 (0)	4 (4.1)	12 (12.2)	82 (83.7)	
PNI						<0.001
Negative	76 (39.6)	11 (14.5)	22 (28.9)	13 (17.1)	30 (39.5)	
Positive	116 (60.4)	0 (0)	1 (0.9)	27 (23.3)	88 (75.9)	
AJCC ypT category						<0.001
0	11 (5.7)	11 (100)	0 (0)	0 (0)	0 (0)	
1	20 (10.4)	0 (0)	12 (60)	5 (25)	3 (15)	
2	23 (12)	0 (0)	5 (21.7)	6 (26.1)	12 (52.2)	
3	57 (29.7)	0 (0)	5 (8.8)	18 (31.6)	34 (59.6)	
4	81 (42.2)	0 (0)	1 (1.2)	11 (13.6)	69 (85.2)	
AJCC ypN category						<0.001
0	55 (28.6)	9 (16.4)	13 (23.6)	17 (30.9)	16 (29.1)	
1	36 (18.8)	0 (0)	6 (16.7)	7 (19.4)	23 (63.9)	
2	50 (26)	0 (0)	3 (6)	10 (20)	37 (74)	
3	51 (26.6)	2 (3.9)	1 (2)	6 (11.8)	42 (82.4)	
AJCC stage						<0.001
0	9 (4.7)	9 (100)	0 (0)	0 (0)	0 (0)	
1	24 (12.5)	0 (0)	14 (58.3)	7 (29.2)	3 (12.5)	
2	53 (27.6)	2 (3.8)	6 (11.3)	14 (26.4)	31 (58.5)	
3	106 (55.2)	0 (0)	3 (2.8)	19 (17.9)	84 (79.2)	
R0 resection						0.212
Yes	159 (82.8)	11 (6.9)	20 (12.6)	35 (22)	93 (58.5)	
No	33 (17.2)	0 (0)	3 (9.1)	5 (15.2)	25 (75.8)	
Adjuvant chemotherapy						0.69
Not received	59 (30.7)	3 (5.1)	6 (10.2)	10 (16.9)	40 (67.8)	
Received	133 (69.3)	8 (6)	17 (35.3)	30 (22.6)	78 (58.6)

**Table 5:** Relationships between clinicopathological characteristics and pathological response evaluated by China-TRG system

Characteristics	Total cases (*n* = 192), no. (%)	China-TRG			*P* value
		Severe response (*n* = 34), no. (%)	Moderate response (*n* = 57), no. (%)	Mild response (*n* = 101), no. (%)	
Gender					0.063
Male	139 (72.4)	30 (21.6)	41 (29.5)	68 (48.9)	
Female	53 (27.6)	4 (7.5)	16 (30.2)	33 (62.3)	
Age					0.820
< 55y	89 (46.4)	15 (16.9)	25 (28.1)	49 (55.1)	
≥ 55y	103 (53.6)	19 (18.4)	32 (31.1)	52 (50.5)	
Location					0.906
Esophagogastric junction	44 (22.9)	6 (13.6)	14 (31.8)	24 (54.5)	
Proximal gastric	71 (37)	14 (19.7)	22 (31)	35 (49.3)	
Distal gastric	77 (40.1)	14 (18.2)	21 (27.3)	42 (54.5)	
Maximal diameter of tumor bed					<0.001
< 4.5 cm	108 (56.2)	28 (25.9)	32 (29.6)	48 (44.4)	
4.5–8 cm	64 (33.3)	5 (7.8)	21 (32.8)	38 (59.4)	
> 8 cm	20 (10.4)	1 (5)	4 (20)	15 (75)	
Histological differentiation					0.001
Well-moderate	45 (23.4)	16 (35.6)	12 (26.7)	17 (37.8)	
Poor	147 (76.6)	18 (12.2)	45 (30.6)	84 (57.1)	
Laurén classification					0.264
Intestinal	77 (40.1)	18 (23.4)	22 (28.6)	37 (48.1)	
Diffuse	73 (38)	13 (17.8)	20 (27.4)	40 (54.8)	
Mixed	42 (21.9)	3 (7.1)	15 (35.7)	24 (57.1)	
LVI					<0.001
Negative	94 (49)	30 (31.9)	35 (37.2)	29 (30.9)	
Positive	98 (51)	4 (4.1)	22 (22.4)	72 (73.5)	
PNI					<0.001
Negative	76 (39.6)	33 (43.4)	20 (26.3)	23 (30.3)	
Positive	116 (60.4)	1 (0.9)	37 (31.9)	78 (67.2)	
AJCC ypT category					<0.001
0	11 (5.7)	11 (100)	0 (0)	0 (0)	
1	20 (10.4)	12 (60)	5 (25)	3 (15)	
2	23 (12)	5 (21.7)	8 (34.8)	10 (43.5)	
3	57 (29.7)	5 (8.8)	26 (45.6)	26 (45.6)	
4	81 (42.2)	1 (1.2)	18 (22.2)	62 (76.5)	
AJCC ypN category					<0.001
0	55 (28.6)	22 (40)	18 (32.7)	15 (27.3)	
1	36 (18.8)	5 (13.9)	12 (33.3)	19 (52.8)	
2	50 (26)	4 (13.9)	19 (38)	27 (54)	
3	51 (26.6)	3 (5.9)	8 (15.7)	40 (78.4)	
AJCC stage					<0.001
0	9 (4.7)	9 (100)	0 (0)	0 (0)	
1	24 (12.5)	14 (58.3)	7 (29.2)	3 (12.5)	
2	53 (27.6)	7 (13.2)	17 (32.1)	29 (54.7)	
3	106 (55.2)	4 (3.8)	33 (31.1)	69 (65.1)	
R0 resection					0.1
Yes	159 (82.8)	31 (19.5)	49 (30.8)	79 (49.7)	
No	33 (17.2)	3 (9.1)	7 (21.2)	23 (69.7)	
Adjuvant chemotherapy					0.975
Not received	59 (30.7)	10 (16.9)	17 (28.8)	32 (54.2)	
Received	133 (69.3)	24 (18)	39 (29.3)	70 (52.6)

**Table 6:** Relationships between clinicopathological characteristics and pathological response evaluated by Becker-TRG system

Characteristics	Total cases (*n* = 192), no. (%)	Becker-TRG				*P* value
		1a (*n* = 11), no. (%)	1b (*n* = 23), no. (%)	2 (*n* = 67), no. (%)	3 (*n* = 91), no. (%)	
Gender						0.142
Male	139 (72.4)	10 (7.2)	20 (14.4)	45 (32.4)	64 (46)	
Female	53 (27.6)	1 (1.9)	3 (5.7)	22 (41.5)	27 (50.9)	
Age						0.945
< 55y	89 (46.4)	6 (6.7)	10 (11.2)	31 (34.8)	42 (47.2)	
≥ 55y	103 (53.6)	5 (4.9)	13 (12.6)	36 (35)	49 (47.6)	
Location						0.562
Esophagogastric junction	44 (22.9)	1 (2.3)	4 (9.1)	15 (34.1)	24 (54.5)	
Proximal gastric	71 (37)	7 (9.9)	8 (11.3)	24 (33.8)	32 (45.1)	
Distal gastric	77 (40.1)	3 (3.9)	11 (14.3)	28 (36.4)	35 (45.5)	
Maximal diameter of tumor bed						0.003
< 4.5 cm	108 (56.2)	9 (8.3)	20 (18.5)	35 (32.4)	44 (40.7)	
4.5–8 cm	64 (33.3)	1 (1.6)	3 (4.7)	28 (43.8)	32 (50)	
> 8 cm	20 (10.4)	1 (5)	0 (0)	4 (20)	15 (75)	
Histological differentiation						0.003
Well-moderate	45 (23.4)	4 (8.9)	12 (26.7)	14 (31.1)	15 (33.3)	
Poor	147 (76.6)	7 (4.8)	11 (7.5)	53 (36.1)	76 (51.7)	
Laurén classification						0.127
Intestinal	77 (40.1)	4 (5.2)	14 (18.2)	25 (32.5)	34 (44.2)	
Diffuse	73 (38)	7 (9.6)	5 (6.8)	24 (32.9)	37 (50.7)	
Mixed	42 (21.9)	0 (0)	4 (9.5)	18 (42.9)	20 (47.6)	
LVI						<0.001
Negative	94 (49)	11 (11.7)	19 (20.2)	39 (41.5)	25 (26.6)	
Positive	98 (51)	0 (0)	4 (4.1)	28 (28.6)	66 (67.3)	
PNI						<0.001
Negative	76 (39.6)	11 (14.5)	22 (28.9)	22 (28.9)	21 (27.6)	
Positive	116 (60.4)	0 (0)	1 (0.9)	45 (38.8)	70 (60.3)	
AJCC ypT category						<0.001
0	11 (5.7)	11 (100)	0 (0)	0 (0)	0 (0)	
1	20 (10.4)	0 (0)	12 (60)	5 (25)	3 (15)	
2	23 (12)	0 (0)	5 (21.7)	10 (43.5)	8 (34.8)	
3	57 (29.7)	0 (0)	5 (8.8)	29 (50.9)	23 (40.4)	
4	81 (42.2)	0 (0)	1 (1.2)	23 (28.4)	57 (70.4)	
AJCC ypN category						<0.001
0	55 (28.6)	9 (16.4)	13 (23.6)	19 (34.5)	14 (25.5)	
1	36 (18.8)	0 (0)	6 (16.7)	16 (44.4)	14 (38.9)	
2	50 (26)	0 (0)	3 (6)	21 (42)	26 (52)	
3	51 (26.6)	2 (3.9)	1 (2)	11 (21.6)	37 (72.5)	
AJCC stage						<0.001
0	9 (4.7)	9 (100)	0 (0)	0 (0)	0 (0)	
1	24 (12.5)	0 (0)	14 (58.3)	7 (29.2)	3 (12.5)	
2	53 (27.6)	2 (3.8)	6 (11.3)	21 (39.6)	24 (45.3)	
3	106 (55.2)	0 (0)	3 (2.8)	39 (36.8)	64 (60.4)	
R0 resection						0.234
Yes	159 (82.8)	11 (6.9)	20 (12.6)	57 (35.8)	71 (44.7)	
No	33 (17.2)	0 (0)	3 (9.1)	10 (30.3)	20 (60.6)	
Adjuvant chemotherapy						0.912
Not received	59 (30.7)	3 (5.1)	6 (10.2)	20 (33.9)	30 (50.8)	
Received	133 (69.3)	8 (6)	17 (35.3)	47 (35.3)	61 (45.9)	

### Tumor regression assessment

We assessed 192 patients using the five TRG systems respectively. According to Mandard-TRG system, there were 11 patients in grade 1, 23 in grade 2, 40 in grade 3, 78 in grade 4, and 40 in grade 5. According to JGCA-TRG system, there were 11 patients in grade 3, 63 in grade 2, 45 in grade 1b, 33 in grade 1a, and 40 in grade 0. According to CAP-TRG system, there were 11 patients in grade 0, 23 patients in grade 1, 40 patients in grade 2, and 118 patients in grade 3. According to China-TRG system, there were 34 patients in severe response grade, 57 in moderate response grade, and 101 in mild response grade. According to Becker-TRG system, there were 11 patients in grade 1a, 23 patients in grade 1b, 67 patients in grade 2, and 91 patients in grade 3 (Tables 2, 3, 4, 5 and 6).

### Correlation between tumor regression and clinicopathological features

Tumor regression evaluated by all the five grading systems has been found significantly associated with histological differentiation, postsurgical T category, postsurgical N category, AJCC stage, LVI, PNI and tumor size (*P* < 0.05) (Tables 2, 3, 4, 5 and 6).

### Survival analysis

One hundred and eighty-one patients were analyzed in survival analysis. At the final follow-up, 81 patients (44.8%) were alive with no evidence of recurrence, while 16 (8.8%) were alive with recurrence. Eighty-three patients (45.9%) had died due to disease recurrence, while 1 (0.5%) had died of unknown reason excluding disease recurrence.

**Fig. 1:**
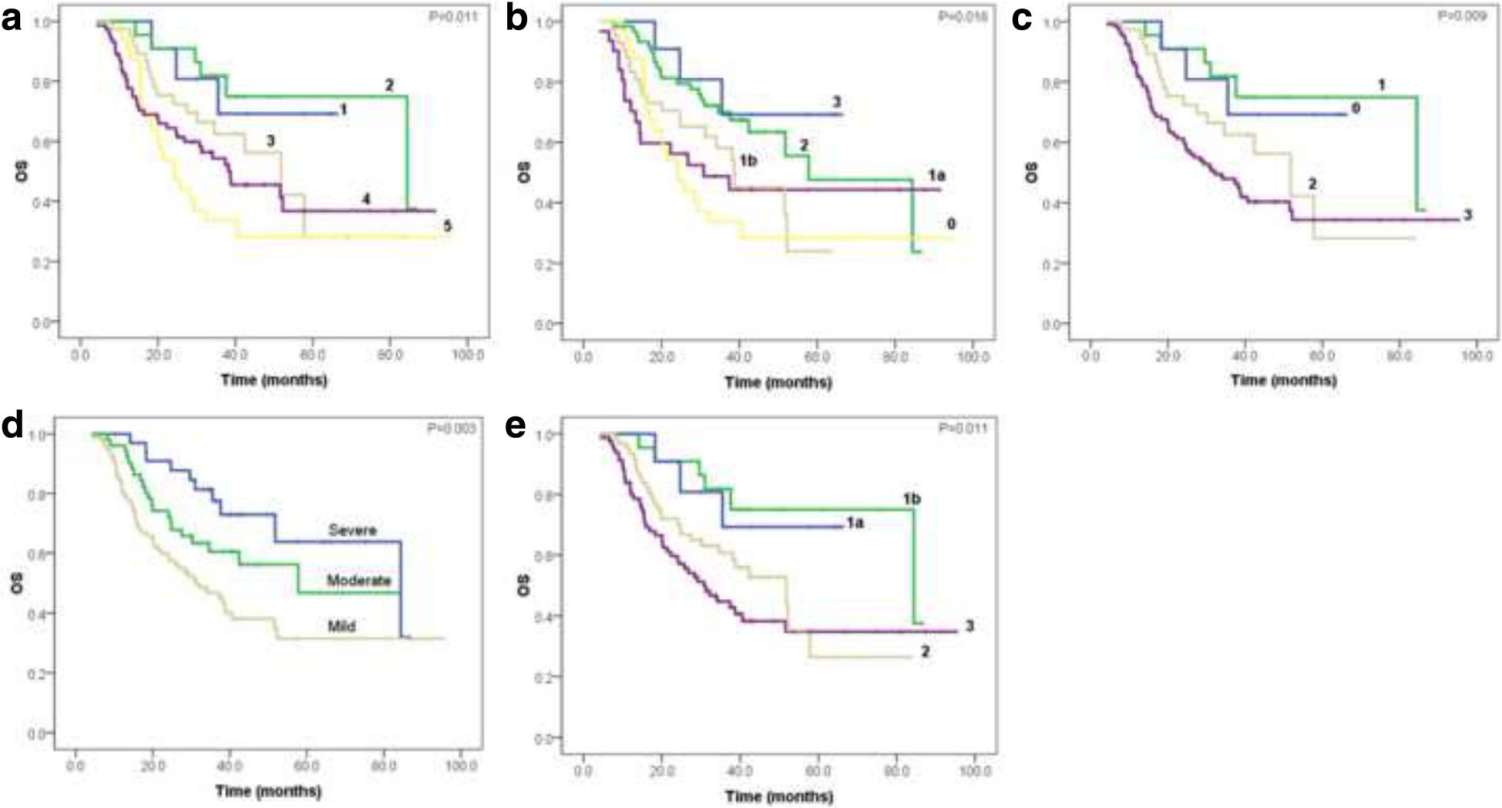
Overall survival curves of five TRG systems, respectively. **a** Mandard-TRG, **b** JGCA-TRG, **c** CAP-TRG, **d** China-TRG and **e** Becker-TRG

**Fig. 2:**
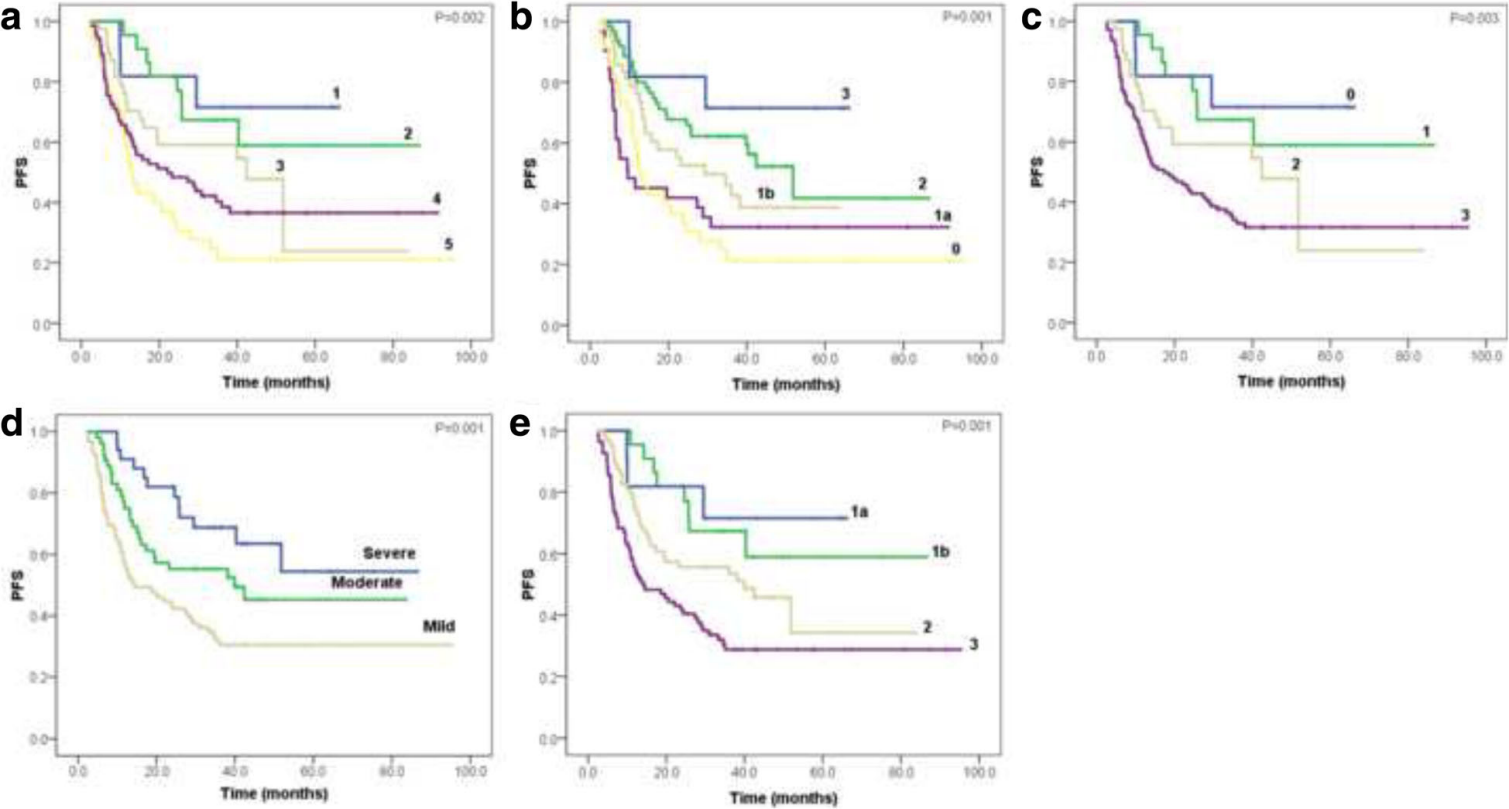
Progression-free survival curves of five TRG systems, respectively. **a** Mandard-TRG, **b** JGCA-TRG, **c** CAP-TRG, **d** China-TRG and **e** Becker-TRG

All of the five TRG systems, histological differentiation, postsurgical T category, postsurgical N category, AJCC stage, LVI, PNI, Laurén classification, R0 resection and tumor size were significant (*P* < 0.05) correlated with OS and PFS in univariate Cox regression analyses (Table 7). The overall and progression-free survival curves of five TRG systems were present in Figs. 1 and 2, respectively.

**Table 7:** Univariate Cox regression analyses

Characteristics	OS			PFS		
	HR	95% CI	*P* value	HR	95% CI	*P* value
Gender						
Male (reference)	1			1		
Female	1.179	0.740–1.876	0.488	1.309	0.859–1.994	0.211
Age						
< 55y (reference)	1			1		
≥ 55y	1.120	0.903–1.390	0.303	0.856	0.577–1.270	0.441
Location						
Esophagogastric junction (reference)	1			1		
Proximal gastric	0.952	0.555–1.632	0.857	0.829	0.506–1.358	0.456
Distal gastric	0.634	0.360–1.117	0.115	0.631	0.380–1.046	0.074
Maximal diameter of tumor bed	1.712	1.275–2.299	<0.001	1.709	1.304–2.239	<0.001
Histological differentiation	3.057	1.617–5.782	0.001	2.667	1.513–4.699	0.001
Laurén classification						
Intestinal (reference)	1			1		
Diffuse	2.228	1.339–3.709	0.002	1.896	1.199–2.999	0.006
Mixed	2.206	1.216–4.003	0.009	1.911	1.113–3.280	0.019
LVI	3.318	2.089–5.270	<0.001	3.324	2.180–5.069	<0.001
PNI	2.878	1.750–4.733	<0.001	2.724	1.737–4.271	<0.001
AJCC ypT category	1.876	1.457–2.415	<0.001	1.739	1.396–2.168	<0.001
AJCC ypN category	1.946	1.572–2.408	<0.001	1.918	1.578–2.330	<0.001
AJCC stage	2.484	1.724–3.580	<0.001	2.270	1.650–3.122	<0.001
Mandard-TRG						
1 (reference)	1			1		
2	0.98	0.244–3.929	0.977	1.293	0.343–4.877	0.704
3	1.956	0.568–6.730	0.287	2.143	0.631–7.283	0.222
4	2.717	0.836–8.831	0.096	3.155	0.979–10.168	0.054
5	3.682	1.094–12.394	0.035	4.570	1.380–15.132	0.013
JGCA-TRG						
3 (reference)	1			1		
2	1.531	0.457–5.126	0.49	1.782	0.539–5.892	0.344
1b	2.626	0.781–8.831	0.119	2.621	0.786–8.738	0.117
1a	2.875	0.836–9.884	0.094	4.022	1.199–13.491	0.024
0	3.676	1.092–12.371	0.035	4.556	1.376–15.085	0.013
CAP-TRG						
0 (reference)	1			1		
1	0.979	0.244–3.927	0.977	1.292	0.343–4.873	0.705
2	1.953	0.568–6.719	0.288	2.140	0.630–7.272	0.223
3	2.996	0.937–9.586	0.064	3.558	1.119–11.311	0.032
China-TRG						
Severe response (reference)	1			1		
Moderate response	1.719	0.808–3.657	0.160	1.661	0.834–3.308	0.149
Mild response	2.856	1.450–5.628	0.002	2.868	1.544–5.328	0.001
Becker-TRG						
1a (reference)	1			1		
1b	0.980	0.244–3.927	0.977	1.292	0.343–4.872	0.705
2	2.216	0.673–7.294	0.190	2.295	0.702–7.502	0.169
3	3.109	0.964–10.028	0.058	3.964	1.239–12.681	0.020
R0 resection						
Yes (reference)	1			1		
No	3.382	2.022–5.659	<0.001	3.656	2.315–5.774	<0.001
Adjuvant chemotherapy						
Not received (reference)	1			1		
Received	1.040	0.633–1.707	0.878	1.227	0.774–1.943	0.384

Postsurgical T category, postsurgical N category, R0 resection and LVI were independent predictors for OS, while LVI, postsurgical N category and R0 resection were independent predictors for PFS, revealed by backward multivariate Cox regression models (Table 8). None of the five tumor regression grading systems were found statistically significant in multivariate survival analysis. In Mandard-TRG system using univariate Cox analysis, the hazard ratio of no response grade was 3.682 and 4.57 in OS and PFS, respectively. In JGCA-TRG system using univariate Cox analysis, the hazard ratio of no response grade was 3.676 and 4.556 in OS and PFS, respectively (Table 7). In the Kaplan-Meier analysis, the median survival time of OS was 84.4 months for patients with Grade 2 in Mandard-TRG system, and 57.8 months for those with Grade 2 in JGCA-TRG system (Table 9).

**Table 8:** Multivariate Cox regression analyses

Characteristics	OS			PFS		
	HR	95% CI	*P* value	HR	95% CI	*P* value
Histological differentiation	-	-	-	1.662	0.914–3.022	0.096
LVI	1.651	0.976–2.793	0.062	1.766	1.091–2.861	0.021
AJCC ypT category	1.355	1.029–1.784	0.031	-	-	-
AJCC ypN category	1.487	1.168–1.894	0.001	1.479	1.180–1.855	<0.001
R0 resection	2.386	1.398–4.073	0.001	2.457	1.516–3.985	<0.001

**Table 9:** Comparison of median survival time between Mandard-TRG and JGCA-TRG

TRG systems	Median for survival time	
	OS	PFS
Mandard-TRG		
1	Not reached	Not reached
2	84.4	Not reached
3	51.8	42.4
4	38.7	22.1
5	24.3	12.8
JGCA-TRG		
3	Not reached	Not reached
2	57.8	51.8
1b	38.7	29.3
1a	30.8	9.6
0	24.3	12.8

## Discussion

Neoadjuvant and adjuvant therapy have been applied to improve the outcome of localized advanced GC, especially in east Asia [11]. Preoperative therapy promoted R0 resection rates in some randomized studies [12]. Although the efficacy of preoperative chemotherapy could be partially reflected by the Response Evaluation Criteria in Solid Tumors (RECIST), RECIST is not always consistent with histopathological regression and prognosis. Thus the relationship among histopathological tumor regression evaluation, efficiency of the multimodality therapy and prognosis requires further illumination. Globally, there have been many kinds of histopathological tumor regression grading systems. Mandard et al. first published their five-tiered TRG system for esophageal carcinoma in 1994. It was reproducible and used widely in carcinomas of esophagus/esophagogastric junction and rectum, but there have been no published applications in GC yet. CAP recommended a simplified four-tiered TRG system based on Mandard-TRG system. In China, a three-tiered grading system is used to assess therapeutic response for solid malignancies. However, its applicability on GC remained unclear. Becker et al. proposed a semi-quantitative four-tiered TRG system in 2003, and then they proved the applicability on GC in 2011. In Japan, the wildly used method to evaluate pathological response is JGCA-TRG, of which the criteria for tumor regression separation are quite distinct from the other four TRG systems.

In this study, 118 (61.4%) patients had mild or minimal tumor regression. Only 11 (5.7%) cases got complete regression without any residual tumor cells on the primary sites, but unfortunately 2 of them were found with residual lymph nodes metastasis, probably resulting in poor outcomes. Twenty-three (12.0%) patients had nearly complete regression with a few residual tumor cells. We supposed the patients who reached complete response should have a better prognosis while the patients who reached nearly complete response should have a worse prognosis. However the actual results did not confirm this. Agoston et al. defined pathological complete response as neither residual primary tumor nor residual lymph node metastasis existing [13]. They reviewed esophageal adenocarcinoma 93 cases with complete response and found that adequacy of histological examination of the tumor bed affected the prognosis. In our study, the number of tumor blocks ranged from 4 to 53 in the complete response cases, while the number ranged from 5 to 77 in the nearly complete response cases. The fewer blocks of some cases might indicate potentially insufficient tumor sampling. Meanwhile, in some other studies which emphasized on adequacy of gross sampling, the percentage of complete response in GC ranged from 1.2 to 3.6% [14–16]. Among the 23 nearly complete response cases in our study, only 5 were classified to postsurgical T3 or T4 categories, while the others were suspected to have earlier T categories before the preoperative therapy. This could be supposed to explain the different prognosis between patients who reached complete response and the patients who had nearly complete response. It is controversial on whether separating the complete response from the nearly complete response. Becker et al. separated the complete response category from the nearly complete response category and assessed separately, however, they combined them for survival analysis [9].

Chirieac et al. demonstrated their TRG system as an independent predictor on esophageal and esophagogastric junction cancer. They evaluated the residual tumor semi-quantitatively as 0% residue, 1–50% residue and >50% residue [17]. Becker et al. found the significance in multivariate analysis on the proportion of residual tumor between <10 and >10% in GC [9]. Both studies were based on large number of patients and long-term follow-up. On contrary, more studies did not demonstrate the independent role of TRG for prognosis. In our univariate survival analysis, all the five TRG systems showed statistical significance which was coincident with other studies [12, 14, 18, 19]. We collected exhaustive clinicopathological characteristics to establish the reliability of this study. The results indicated that all the TRG systems tightly correlated with LVI, postsurgical T and N categories, therefore the staging status and LVI would affect the statistical significance of the TRG systems in multivariate survival analysis. This could elucidate the absence of independent significance of the TRG systems. As compared with other grading systems, the hazard ratio of no/slightly response grade in both Mandard-TRG system and JGCA-TRG system revealed higher hazard of death and disease progression than that of severe response grade when using univariate Cox survival analysis. Furthermore, the main difference between the two five-tiered TRG systems is whether separating the category of nearly complete response from partial response. In Mandard-TRG system, the category of nearly complete response is separated, however, in JGCA-TRG system, it is not. Because the median survival time of patients with nearly complete response in Mandard-TRG system (84.4 months) was much longer than those with partial response in JGCA-TRG (57.8 months) (Table 9), separation of nearly complete response and partial response categories in Mandard-TRG system could be more reasonable for prognosis prediction.

## Conclusions

This study analyzed five classic TRG systems on GC after neoadjuvant chemotherapy and revealed the significance of all the five TRG systems in univariate survival analysis. We recommend Mandard-TRG system in GC evaluation for prediction of survival.

## Abbreviations

AJCC: American Joint Committee on Cancer
CAP: College of American Pathologists
GC: Gastric cancer
JGCA: The Japanese Gastric Cancer Association
LVI: Lymph-vascular invasion
OS: Overall-survival
PFS: Progression-free-survival
PNI: Perineural invasion
RECIST: Response Evaluation Criteria in Solid Tumors
TRG: Tumor regression grading
